# Growth Performance and Economic Efficiency of SASSO‐C44 Broilers Fed on Diets Containing Varying Levels of Dried Blood–Rumen Mixture as a Substitute for Roasted Soybean Meal

**DOI:** 10.1002/fsn3.71678

**Published:** 2026-03-24

**Authors:** Soressa Shuma, Demissu Hundie Senbeta, Malatji Dikeledi Petunia

**Affiliations:** ^1^ Department of Agriculture and Animal Health, College of Agriculture and Environmental Science University of South Africa Johannesburg South Africa; ^2^ Department Animal Science, College of Agriculture and Natural Resources Dambi Dollo University Dambi Dollo Ethiopia; ^3^ Department of Animal Science, Faculty of Agriculture, Shambu Campus Wollega University Shambu Ethiopia

**Keywords:** DBRCM, economic efficiency, performance index, roasted soybean meal

## Abstract

This study evaluated the effects of partially replacing roasted soybean meal (RSBM) with a locally processed dried blood–rumen content mixture (DBRCM) in broiler diets on growth performance and economic efficiency. A total of 200 SASSO‐C44 broiler chicks were randomly allocated to five dietary treatments in a completely randomized design. The treatments consisted of graded inclusion levels of DBRCM (0%, 20%, 40%, and 60%) and a commercial feed used as a control. During the starter phase, dry matter intake (DMI) and body weight gain (BWG) declined significantly as the level of DBRCM in the diet increased (*p* < 0.05). Birds fed higher DBRCM inclusion levels (T3 and T4) recorded significantly lower mean daily DMI (33.92 and 33.17 g/bird, respectively) compared with those receiving the control and lower inclusion diets (T1: 37.05 g/bird; T5: 37.45 g/bird). A similar trend was observed for final body weight, which decreased from 771.5 g in T1 to 732.3 g in T3, with a slight improvement in T5. However, over the entire 56‐day experimental period, final body weight, feed conversion ratio (FCR), and overall performance index did not differ significantly among treatments (*p* ≥ 0.05). Economic analysis showed that diets containing 40% and 60% DBRCM significantly reduced feed cost per kilogram of weight gain, net revenue, and overall economic efficiency (*p* < 0.05). Overall, the findings indicate that DBRCM can serve as a viable and low‐cost alternative protein source in broiler production, contributing to improved sustainability without compromising overall productivity.

## Graphical abstract

This study aimed to investigate the growth performance and economic efficiency of SASSO‐C44 broilers fed diets containing different levels of dried blood–rumen content mixture (DBRCM) instead of roasted soybean meal. Two‐hundred‐day‐old chicks were randomly allocated to five different dietary treatments containing 0%, 20%, 40%, and 60% DBRCM and a commercial feed as a control. In the starter phase, increasing DBRCM in broiler diets significantly decreased dry matter intake and body weight gain of broilers. However, in the whole experimental period of 56 days, it did not significantly affect the final body weight, feed conversion ratio, and performance index of broilers. In addition, the economic analysis of this study revealed that increasing DBRCM in broiler diets significantly reduced the feed cost of weight gain and increased the profit of broiler production. The results of this study indicated that dried blood–rumen content mixture can be considered a sustainable and cost‐effective protein source in broiler diets without affecting broiler performance.

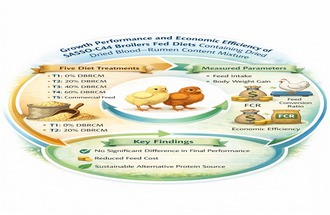

## 1 Introduction

In developing countries, poultry production benefits from low labor and housing costs and favorable climatic conditions for simple housing systems; nevertheless, feed remains the dominant cost component, accounting for about 70%–85% of total production costs (Shuma et al. 2025), largely driven by the high prices of protein‐rich ingredients such as soybean meal, groundnut cake, and fish meal (Beaumont et al. 2022). This economic reality underscores the urgent need to identify locally available, low‐cost alternative protein sources that do not directly compete with human food. Dried blood–rumen content mixture (DBRCM), obtainable from backyard slaughtering sites, slaughter slabs, slaughterhouses, and abattoirs, represents a promising option, particularly in Ethiopia where a large livestock population generates substantial quantities of rumen content and blood annually (Roccabruna et al. 2026). Estimates indicate that slaughtered cattle produce considerable amounts of rumen content dry matter and blood, translating into large volumes of potential feed resources and significant economic value if properly processed and commercialized, with additional benefits in reducing environmental pollution caused by improper disposal of these by‐products (Wang et al. 2025). Recent studies have strengthened the scientific basis for replacing conventional soybean meal with alternative protein sources, showing that partial substitution with agro‐industrial or slaughterhouse by‐products can sustain or improve protein efficiency, growth performance, and metabolic status when diets are nutritionally balanced and appropriately processed, as demonstrated in broiler chickens (Tibebu et al. 2024) and using blood meal as a soybean meal replacer (Gupta et al. 2023). Despite this growing body of evidence, limited recent research has been conducted in Ethiopia, including in Dambi Dollo, on the systematic evaluation of rumen content and blood‐based mixtures such as DBRCM for poultry feeding, thereby leaving a clear and persisting knowledge gap in terms of nutritional value, biological utilization, and economic feasibility under local production conditions (Osei‐Adjei et al. 2025). Therefore, in response to persistent feed cost challenges, environmental concerns, and recent advances in alternative protein utilization, this study was conducted to assess the growth performance and economic efficiency of SASSO‐C44 broiler chickens fed diets containing graded levels of DBRCM as a partial replacement for roasted soybean meal.

## 2 Materials and Methods

### 2.1 Study Area

The study was conducted in Dambi Dollo town, located in the Kellem Wollega Zone of western Oromia, Ethiopia, approximately 642 km west of Addis Ababa. The area lies at an altitude ranging from 1500 to 1740 m above sea level and receives annual rainfall between 850 and 1308 mm, with temperatures ranging from 15°C to 28°C. Dambi Dollo town covers approximately 6160 ha and is situated at an elevation of about 2420 m above sea level.

### 2.2 Strengths and Limitations of the Study

#### 2.2.1 Strengths of the Study

A major strength of this study lies in its strong practical relevance to poultry production under developing‐country conditions. The experimental diets were formulated using locally available feed resources and processed with simple, low‐cost techniques (boiling, sun‐drying, and roasting), enhancing the applicability and scalability of the findings for small‐ and medium‐scale poultry producers. The use of a commercially important dual‐purpose strain (SASSO‐C44) further strengthens the relevance of the results to current production systems in Ethiopia and similar agro‐ecological zones.

Methodologically, the study employed a completely randomized design with adequate replication, isocaloric and isonitrogenous diets, and standardized bird management and health programs, which together minimized confounding effects and improved the reliability of dietary comparisons. In addition, the inclusion of a commercial feed treatment provided a realistic benchmark against industry standards, strengthening the validity of performance and economic interpretations.

Another key strength is the integrated evaluation approach, which combined growth performance, health management, chemical composition analysis, and economic efficiency. This holistic assessment allows conclusions to be drawn not only on nutritional adequacy but also on economic feasibility and sustainability, which are critical factors for feed innovation. Furthermore, laboratory‐based proximate analyses conducted at a national reference institute ensured the credibility and accuracy of nutrient characterization.

#### 2.2.2 Limitations of the Study

Despite these strengths, the study has certain limitations that should be acknowledged. First, the experiment was conducted under controlled on‐station conditions, which may not fully capture the variability in management, hygiene, and environmental stressors commonly encountered in smallholder or semi‐intensive production systems. Therefore, caution is warranted when extrapolating the findings directly to all production settings.

Second, while growth performance and economic indicators were comprehensively evaluated, the study did not assess nutrient digestibility, amino acid availability, or gut health responses, which could provide deeper physiological explanations for the observed outcomes. In particular, the variable composition of rumen content and blood—depending on animal diet and slaughter conditions—may influence nutrient consistency, and this variability was not chemically standardized beyond proximate analysis.

Third, the processing methods relied on sun‐drying, which, although practical and cost‐effective, may be influenced by weather conditions and could lead to variation in drying efficiency and microbial safety if not carefully managed. Additionally, the study focused on a single mixing ratio (1:1) of dried blood to rumen content, limiting conclusions about the optimal proportion of these two components.

Finally, the duration of the study was limited to the starter and finisher phases, and no post‐slaughter carcass traits or meat quality parameters were evaluated, which are important considerations for market acceptance and consumer preference.

#### 2.2.3 Implications for Future Research

Future studies should validate these findings under on‐farm conditions, include digestibility and amino acid profiling, and explore alternative processing and mixing ratios to optimize nutritional consistency. Investigations into gut microbiota response, carcass characteristics, and long‐term health implications would further strengthen the scientific and practical foundation for widespread adoption of DBRCM in poultry feeding systems.

### 2.3 Blood Collection and Processing

Fresh cattle blood was collected immediately after slaughter. To reduce microbial contamination and moisture content, the blood was boiled at 100°C for 45 min. The boiled blood was poured onto clean plastic sheets to prevent contamination and sun‐dried for 3–5 days, depending on weather conditions and material thickness. The dried blood was ground using a hand mortar and sieved through a 3‐mm mesh to achieve uniform particle size comparable to commercial broiler feed.

### 2.4 Rumen Content Collection and Processing

Fresh rumen contents were collected from cattle slaughtered at the Dambi Dollo University municipal abattoir. The rumen material was boiled for 2 h in a metallic vat placed over an open fire, with occasional stirring to reduce microbial load. The boiled material was spread on sanitized plastic sheets and sun‐dried for 3–4 days. Oversized particles were crushed using a hand mortar and passed through a 3‐mm sieve to obtain uniform particle size.

### 2.5 Preparation of Dried Blood–Rumen Content Mixture

Dried blood and dried rumen content were thoroughly mixed in a 1:1 ratio to produce DBRCM, which was incorporated into broiler diets at different inclusion levels as a replacement for roasted soybean meal.

### 2.6 Roasted Soybean Meal Preparation

Full‐fat soybeans were purchased from the Dambi Dollo local market. The seeds were cleaned by windowing and hand‐picking, washed with water, and roasted on a metal tray for 15 min with frequent stirring until a golden‐brown color was achieved. Roasting was performed to deactivate antinutritional factors such as trypsin inhibitors. The roasted soybeans were then ground and sieved through a 3‐mm mesh to produce roasted soybean meal for diet formulation.

### 2.7 Experimental Birds and Management

Two‐hundred‐day‐old SASSO‐C44 broiler chicks were obtained from Ethio Chicken PLC and randomly allocated to experimental pens using a completely randomized design (CRD). Twenty pens (1.8 m^2^ each; 0.12 m^2^ per bird) constructed of wood and wire mesh were used. The pens were cleaned, disinfected with 37% formalin 2 weeks prior to chick placement, and allowed to dry.

Each pen was bedded with dried straw to a depth of 8 cm. Brooding temperature was maintained at 35°C–32°C during the first week and reduced by 2°C weekly thereafter using 200‐W bulbs. Adequate ventilation was ensured through window openings. Birds were provided feed and fresh water ad libitum. Vaccinations against Newcastle disease (HB1 and Lasota) were administered on days 7, 21, and 45; Gumboro disease on days 14, 28, and 42; and fowl typhoid on day 49. Amprolium was administered prophylactically against coccidiosis.

### 2.8 Experimental Design and Diets

Experimental diets were formulated using maize, wheat middling's, noug seed cake, roasted soybean meal, DBRCM, fish meal, vitamin premix, lysine, methionine, salt, and limestone. All ingredients were ground to pass through a 3‐mm sieve.

Diets were formulated to be isocaloric and isonitrogenous. Starter diets (1–28 days) contained 3000 kcal ME/kg DM and 23% crude protein, while finisher diets (29–56 days) contained 3200 kcal ME/kg DM and 20% crude protein. Treatments were as follows:

T1: 100% roasted soybean meal (control).

T2: 80% RSBM + 20% DBRCM.

T3: 60% RSBM + 40% DBRCM.

T4: 40% RSBM + 60% DBRCM.

T5: commercial feed.

### 2.9 Data Collection

Feed intake was recorded daily, and dry matter intake was calculated. Birds were weighed weekly to determine body weight gain, average daily gain, feed conversion ratio, and performance index. Mortality was recorded throughout the experiment.

### 2.10 Chemical Analysis

Representative samples of feed ingredients, diets, and refusals were analyzed for dry matter, crude protein, ether extract, crude fiber, and ash content at the National Veterinary Institute, Bishoftu, Ethiopia. Metabolizable energy was estimated using standard equations.

### 2.11 Economic Analysis

Economic efficiency was evaluated based on feed cost per kilogram of weight gain, total revenue, net revenue, economic efficiency, and relative economic efficiency.

### 2.12 Statistical Analysis

Data was analyzed using ANOVA with the General Linear Model procedure of SAS version 9.4. Treatment means were separated using Tukey's test, and significance was declared at *p* < 0.05.

### 2.13 Novelty and Knowledge Gap

Though some research work has been conducted on the nutritional value of rumen contents or blood meal as an alternative poultry feed, most of these studies were carried out on a small scale, at a single inclusion level, or using nonlocal broiler strains. Furthermore, most of these studies did not consider the biological and economic efficiency of the feed supplementations simultaneously. In Ethiopia, most of the studies conducted on rumen contents or blood meal as poultry feed supplements have been limited to the use of these two products separately, with little emphasis on the joint use of the two products as a dried blood–rumen content mixture (DBRCM), which has a balanced protein profile and better amino acid availability. Additionally, there is a lack of empirical information on the graded replacement levels of roasted soybean meal with DBRCM in actual production settings, especially for SASSO‐C44 broiler chickens, which are widely recommended for semi‐intensive production in Ethiopia.

Moreover, it is also important to note that in most of the previous studies, there was a lack of consideration of environmental sustainability factors, such as waste reduction and abattoir by‐product valorization, in addition to economic performance indicators for small‐ and medium‐scale poultry farmers. Therefore, this study fills these important research gaps by examining the effects of various levels of DBRCM implementation on the growth performance, feed efficiency, and economic returns of SASSO‐C44 broilers. By making connections between animal performance, feed cost savings, and environmental waste management in the local Ethiopian setting, this study presents new and location‐specific evidence to support the sustainable use of slaughterhouse by‐products as alternative protein sources in poultry production systems.

## 3 Results

The chemical composition of the ingredients, including blood meal, dried rumen content, and DBRCM, is presented in Table 1. Blood and rumen contents were collected immediately after slaughter, processed separately, and combined in a 1:1 ratio before chemical analysis at the National Veterinary Institute, Bishoftu, Ethiopia.

**TABLE 1:** Chemical composition of feed ingredients used for preparing experimental diets as DM basis.

No	Feed type	DM%	Ash (MM)%	CF%	CP%	EE%	Ca%	NFE%	ME (kcal/kg DM)
1	DBRCM	88.00	11.29	18.18	44.17	1.43	2.09	24.93	2636.17
2	NSC	96.00	4.97	29.06	33.68	9.52	1.85	22.78	2833.31
3	Maize	90.23	5.25	4.99	8.40	1.93	1.85	79.44	3288.66
4	S. Bean	96.07	5.55	17.80	40.41	5.98	1.88	30.26	3058.30
5	Wheat	91.37	1.86	2.52	11.55	2.18	1.11	81.89	3512.77
6	Fish meal	94.97	22.32	4.42	51.20	3.60	8.66	18.45	2844.12

Abbreviations: Ca, calcium; CF, crude fiber; CP, crude protein; DBRCM, dried blood–rumen content mixture; DM, dry matter; EE, ether extract; kg, kilogram; ME, metabolizable energy; MM, mineral matter; NFE, nitrogen free extract; NSC, noug seed cake.

### 3.1 Chick Starter and Finisher Ration Feed Ingredients

The feed ingredients and chemical composition of the five dietary treatment groups of starter and finisher ration are presented in Table 2.

**TABLE 2:** Proportion of ingredients used in formulating broiler starter and finisher rations, and chemical composition of the treatment groups.

Ingredients %	For broilers starters					For broilers finisher				
Feed ingredients	T1	T2	T3	T4	T5 (control)	T1	T2	T3	T4	T5 (control)
Soyabean	30	24	18	12		30	24	18	12	
DBRMC	0	6	12	18		0	6	12	18	
NCS	15.7	15.1	14.5	12.4		2.5	2	1.6	1.2	
Fishmeal	2.4	2.2	2.1	2.5		2	1.9	1.2	0.8	
Maize	39.5	40.4	40.8	39.9		47.9	49.1	49.4	50.1	
Wheat	9.3	9.2	9.5	12.1		14.5	13.9	14.7	14.8	
Limestone	1.2	1.2	1.2	1.2		1.2	1.2	1.2	1.2	
Vitamin premix	1	1	1	1		1	1	1	1	
Salt	0.5	0.5	0.5	0.5		0.5	0.5	0.5	0.5	
Lysine	0.2	0.2	0.2	0.2		0.2	0.2	0.2	0.2	
Methionine	0.2	0.2	0.2	0.2		0.2	0.2	0.2	0.2	
Total	100	100	100	100		100	100	100	100	
DM (%)	89.43	88.95	87.68	87.45	89.3	90.06	90.2	89.3	89.5	89.87
CP (% DM)	23.01	23.05	23.08	23.1	23	19.68	20.05	19.75	20.1	20.03
ME (kcal/Kg DM)	3073	3049	3021	3012	3084	3285	3264	3236	3210	32,086
CF (% DM)	4.7	4.8	4.7	4.4	3.8	2.8	2.2	3	2.9	3
MM (% DM)	11.57	11.28	10.67	10.42	11.4	12.98	12.76	12.33	11.1	12.638
EE (% DM)	7.3	6.6	4.2	3.8	3.6	8.6	7	5.8	4.1	5.5
NFE (% DM)	2775	2704	2560	2499	2744	43.6	44.2	44.3	44.9	47.9
Ca (% DM)	1.27	1.6	1.5	1.4	1.37	0.96	0.98	0.99	1.1	1.3

*Note:* Roasted soybean meal; DBRCM = Dried Blood Rumen Content Mixture; NSC = Noug Seed Cake; T1 = 0% DBRCM + 100% RSBM, T2 = 20% DBRCM + 80% RSBM; T3 = 40% DBRCM + 60% RSBM; T4 = 60% DBRCM + 40% RSBM; T5 = commercial feed.

Abbreviations: Ca, calcium; CF, crude fiber; CP, crude protein; DM, dry matter; EE, ether extract; ME, metabolizable energy; NFE, nitrogen free extract.

### 3.2 Dry Matter Intake, Body Weight Change, and Feed Conversion Ratio of SASSO‐C44 Broiler Chicks

Dry matter intake (DMI), body weight change, and feed conversion ratio (FCR) of SASSO‐C44 broiler chicks fed diets containing graded levels of dried blood–rumen content mixture (DBRCM) as a replacement for roasted soybean meal are presented in Table 3. During the starter phase, birds fed DBRCM‐containing diets exhibited significant differences in daily DMI compared with the control group (*p* < 0.05). The lowest DMI (*p* < 0.05) was recorded in birds receiving the 60% DBRCM diet (T4) during both the finisher phase and the overall experimental period.

**TABLE 3:** Dry matter intake, body weight change, feed conversion ratio and mortality rate of SASSO‐C44 broiler chicks during starter phase (1–28 days), finisher phase (29–56 days), and enter experiment (1–56 days).

Parameters	Experimental diets						
	T1	T2	T3	T4	T5	SEM	*p*
*Starter phase*							
Mean daily DM intake (g/bird)	37.05^bc^	35.13^bc^	33.92^c^	33.17^c^	37.45^a^	0.67	0.003
Mean total DM intake (g/bird)	10.13^a^	9.91^b^	9.44^c^	9.18^d^	10.19^a^	11.49	0.001
Initial body weight (g)	41.5^a^	41.6^a^	41.4^a^	41.3^a^	41.3^a^	0.037	0.24
Mean final body weight (g)	771.5^a^	769.4^a^	732.3^c^	746.4^b^	772.7^a^	22	0.001
Mean daily weight gain (g/bird)	26.0^a^	25.9^a^	24.6^c^	25.1^b^	26.1^a^	0.028	0.001
Mean total gain (g)	730^a^	727.7^a^	690.8^c^	705^b^	731.3^a^	22	0.001
FCR (gDMI I/g gain)	1.56^a^	1.53^a^	1.56^a^	1.49^a^	1.56^a^	0.011	0.083
*Finisher phase*							
Mean daily DM intake (g/bird)	107.6^a^	104.6^ab^	101.2^b^	100.8^b^	108.5^a^	2.98	0.001
Mean total DM intake (g/bird)	2964^b^	2937^c^	2826^e^	2838^d^	2989^a^	9.6	0.007
Initial body weight (g)	771.5^a^	769.4^a^	732.3^c^	746.4^b^	772.7^a^	21.5	0.001
Mean final body weight (g)	1774^a^	1772^a^	1720.6^a^	1713^a^	1807^a^	2963	0.12
Mean daily weight gain (g/bird)	35.8^a^	35.8^a^	35.2^a^	34.5^a^	36.9^a^	3.9	0.54
Mean total gain (g)	1003^a^	1002.6^a^	988.3^a^	966.6^a^	1034.3^a^	3059	0.55
FCR (gDMI I/g gain)	2.96^a^	2.93^ab^	2.87^b^	2.92^ab^	2.88^b^	0.001	0.009
*Entire experimental period*							
Mean daily DM intake (g/bird)	71.6^a^	69.81^b^	67.25^c^	66.37^c^	71.6^a^	0.4	0.001
Mean total DM intake (g/bird)	4010^a^	3908^b^	3764^c^	3717^d^	4009^a^	2.29	0.001
Initial body weight (g)	41.5a	41.6a	41.4a	41.3a	41.3a	0.037	0.24
Mean final body weight (g)	1774^a^	1772.3^a^	1720.6^a^	1713^a^	1807^a^	2963^a^	0.12
Mean daily weight gain (g/bird)	30.9^a^	30.9^a^	29.9^a^	29.8^a^	31.5^a^	0.94	0.12
Mean total gain (g)	1732.5^a^	1730.6^a^	1679^a^	1671^a^	1765.6^a^	2966	0.12
FCR (gDMI I/g gain)	2.29^a^	2.27^a^	2.24^a^	2.22^a^	2.26^a^	0.029	0.984

*Note:* Means with a different superscript in a row are significantly different (*p* < 0.05); T1—0% DBRCM + 100% RSBM; T2—20% DBRCM + 80% RSBM; T3—40% DBRCM + 60% RSBM; T4—60% DBRCM + 40% RSBM; T5—commercial feed.

Abbreviations: DMI, dry matter intake; FCR, feed conversion ratio; g, gram; SEM, standard error of the mean.

Body weight gain was also significantly affected by dietary treatment. During the starter phase, birds fed the 60% DBRCM diet (T4) recorded the lowest body weight gain. Significant differences in body weight gain among treatments were observed during the finisher phase and across the entire experimental period (*p* < 0.05).

Feed conversion ratio was significantly influenced by dietary treatment during both the starter and finisher phases (*p* < 0.05). However, when evaluated over the full 56‐day production cycle, overall FCR did not differ significantly among the treatment groups (*p* ≥ 0.05).

### 3.3 Performance Index and Mortality of Broilers Fed DBRCM‐Based Diets

The performance index of broiler chickens fed diets containing graded levels of dried blood–rumen content mixture (DBRCM) as a substitute for roasted soybean meal is presented in Table 4. No significant differences (*p* ≥ 0.05) were observed in the performance index among the dietary treatments during the starter phase, finisher phase, or over the entire experimental period.

**TABLE 4:** Performance index of broilers fed diets containing graded levels of DBRCM during starter phase (1–28 days), finisher phase (29–56 days), and the entire experimental period (1–56 days).

Performance index	Experimental diets							
	T1	T2	T3	T4	T5	Mean	SEM	*p*
Starter phase	55.31	55.68	53.0	58.21	54.45	55.34	34	0.78
Finisher phase	33.47	34.19	34.28	33.19	36.12	34.22	11.69	0.75
Entire experiment	72.5	74.75	72.0	72	76.25	73.5	4.96	0.055
Number of mortalities	0	2	1	1	0	0.8	0.13	0.001
Mean mortality	0	0.5	0.25	0.25	0	02	0.02	0.02
Mortality %	0	5	2.5	2.5	0	2	2.5	0.02
Survival %	100	95	97.5	97.5	100	98	2.5	0.02

*Note:* T1 = 0% DBRCM + 100% RSBM; T2 = 20% DBRCM + 80% RSBM; T3 = 40% DBRCM + 60% RSBM; T4 = 60% DBRCM + 40% RSBM; T5 = commercial feed. SEM: standard error of the mean performance index (PI) = (body weight in kg/FCR) × 100.

Similarly, mean mortality rate was not significantly affected by dietary treatment (*p* ≥ 0.05), indicating that inclusion of DBRCM had no adverse effect on bird survival.

### 3.4 Economic Efficiency of Experimental Diets

The economic efficiency of experimental diets is presented in Table 5. The cost per kilogram of feed differed significantly among dietary treatments (*p* < 0.05). Diets T1 and T5 were the most expensive on a per‐kilogram basis, whereas T4 was the least expensive (*p* < 0.05).

**TABLE 5:** Economic efficiency of trial rations.

Parameters	Experimental diet							
	T1	T2	T3	T4	T5	Mean	SEM	*p*
Mean final body weight (kg)	1.774^a^	1.772^a^	1.720^a^	1.7013^a^	1.806^a^	1.75	0.2	0.12
Feed cost/kg feed (Birr)	66.45^b^	61.18^c^	55.35^d^	52.38^e^	75.5^a^	62.17	4.87	0.001
Total feed consumed/bird (kg)	4.82^a^	4.73^b^	4.65^b^	4.44^c^	4.66^b^	4.66	0.01	0.001
Feed cost/kg gain (Birr)	168.9^b^	151.03^c^	137.76^d^	129.01^d^	185.47^a^	154	21.026	0.001
Total feed cost (Birr/bird)	299^b^	268^c^	236^d^	221^e^	337^a^	272	25.59	0.001
Total revenue (Birr/bird)	949^ab^	942^ab^	945^ab^	93^0b^	955^a^	942	113	0.02
Net revenue (Birr/bird)	650^b^	675^b^	697^a^	706^a^	619^c^	668	98	0.001
Economic efficiency (EE)	2.17^c^	2.50^b^	2.94^a^	3.19^a^	1.84^d^	2.53	0.01	0.001
Relative economic efficiency (REE)	1.18^c^	1.35^b^	1.6^a^	1.73^a^	1^d^	1.37	0.04	0.001

*Note:* Means with a different superscript in a row are significantly different (*p* < 0.05); T1 = 0% DBRCM + 100% RSBM; T2 = 20% DBRCM + 80% RSBM; T3 = 40% DBRCM + 60% RSBM; T4 = 60% DBRCM + 40% RSBM; T5 = commercial feed; SEM = standard error of the mean; kg = kilogram; total revenue (Birr) = total gain (kg) × price of 1 kg body weight on sale; net revenue = total revenue from the gain − total feed cost (Birr); economic efficiency = net revenue/total feed cost; relative economic efficiency = economic efficiency/control economic efficiency, (Birr = Ethiopian Cash).

Similarly, the cost per kilogram of body weight gain varied significantly among treatments (*p* < 0.05). The highest cost per kilogram of gain was observed in birds fed diets T1 and T5, while the lowest cost was recorded in birds fed diet T4. Significant differences (*p* < 0.05) were also observed in total feed cost across treatments, with birds in T5 incurring the highest total feed cost and those in T4 incurring the lowest.

Economic efficiency differed significantly among dietary treatments (*p* < 0.05). Birds fed diets T3 and T4 exhibited the highest economic efficiency, whereas those fed diets T1 and T5 showed the lowest economic efficiency.

### 3.5 Growth Performance and Feed Conversion Rate

Final body weights of broiler chickens did not differ significantly among dietary treatments (*p* ≥ 0.05), with a mean final weight of 1.75 kg. This finding indicates that substituting roasted soybean meal with dried blood–rumen content mixture (DBRCM) at any of the inclusion levels tested did not adversely affect final growth performance.

In contrast, total feed consumption per bird differed significantly among treatments (*p* = 0.001). Birds fed the T4 diet consumed the least amount of feed (4.44 kg per bird), which was significantly lower (*p* < 0.05) than feed intake recorded for birds in T5 (4.66 kg) and T1 (4.82 kg). However, feed intake of birds fed the T4 diet did not differ significantly (*p* ≥ 0.05) from those fed T2 (4.73 kg) and T3 (4.65 kg).

The highly significant treatment effect indicates that differences in feed intake were not due to random variation but were associated with dietary formulation, particularly the level of DBRCM inclusion, which had a measurable influence on total feed consumption.

### 3.6 Feed Cost Analysis

The cost per kilogram of feed was directly influenced by dietary formulation, particularly the inclusion level of dried blood–rumen content mixture (DBRCM). The control diet (T5), which contained no DBRCM, had the highest feed cost at 75.5 Birr/kg (approximately USD 1.34/kg) and was significantly more expensive than all other diets (*p* < 0.05). Feed cost per kilogram decreased progressively with increasing levels of DBRCM inclusion, with diet T4 recording the lowest cost at 52.38 Birr/kg (approximately USD 0.93/kg).

This inverse relationship between DBRCM inclusion and feed cost was also reflected in the cost per kilogram of body weight gain. Birds fed diets T3 and T4 recorded the lowest and most economical costs per kilogram of gain, at 137.76 Birr/kg (approximately USD 2.44/kg) and 129.01 Birr/kg (approximately USD 2.28/kg), respectively. These values were significantly lower (*p* < 0.05) than those observed for birds fed T1 (168.9 Birr/kg; approximately USD 2.99/kg) and T5 (185.47 Birr/kg; approximately USD 3.28/kg).

Similarly, total feed cost per bird followed the same trend. Diet T4 resulted in the lowest total feed cost per bird (221 Birr; approximately USD 3.91), whereas birds fed the control diet (T5) incurred the highest cost (337 Birr per bird; approximately USD 5.96). Overall, these results clearly demonstrate the substantial cost‐saving potential of incorporating DBRCM into broiler diets, particularly in resource‐limited production systems.

### 3.7 Economic Returns and Efficiency

Although total revenue per bird did not differ significantly among dietary treatments (*p* ≥ 0.05), ranging narrowly from 930 to 955 Birr (approximately USD 16.91–17.36), both net revenue and overall economic efficiency differed significantly (*p* < 0.05). The highest net revenue was obtained from birds fed diet T4 (706 Birr; approximately USD 12.84), followed closely by those fed diet T3 (697 Birr; approximately USD 12.67). These two diets generated substantially higher net returns than the remaining treatments.

In contrast, birds fed the control diet (T5) generated the highest gross revenue but recorded the lowest net revenue (619 Birr; approximately USD 11.25), largely due to the high cost of feed. Economic efficiency (EE) and relative economic efficiency (REE) followed a similar pattern. Diet T4 exhibited the highest efficiency (EE = 3.19; REE = 1.73), which was statistically comparable to T3 but significantly superior to T1, T2, and T5. The control diet (T5) recorded the lowest efficiency values (EE = 1.84; REE = 1.00), indicating comparatively poor economic viability.

## 4 Discussion

This study advances existing knowledge on the use of dried blood–rumen content mixture (DBRCM) in broiler nutrition by moving beyond descriptive performance outcomes to interpret the biological significance of the results. Specifically, it elucidates the physiological and biochemical mechanisms through which DBRCM influences feed intake, growth dynamics, nutrient utilization, and economic efficiency, thereby demonstrating clear progress over earlier studies that primarily reported agreements or discrepancies in performance responses without mechanistic explanation. The observed reduction in dry matter intake (DMI) with increasing DBRCM inclusion—most pronounced in the 60% replacement group (T4) reflects more than simple diet rejection; it aligns with established physiological responses to changes in sensory cues and gastrointestinal signaling. In broilers, feed palatability is strongly influenced by visual and olfactory characteristics, which interact with central appetite regulation pathways (e.g., hypothalamic neuropeptides) to modulate voluntary intake (San‐Miguel et al. 2023). The darker color and stronger odor of high‐DBRCM diets likely triggered early satiety responses through conditioned aversion and altered chemosensory feedback, consistent with findings in poultry nutrition where changes in diet appearance and odor influence intake behavior (Vasluianu et al. 2024).

Moreover, the elevated crude fiber content in DBRCM alters digesta physical properties, increasing bulk and retention time in the gut. This mechanical effect activates mechanoreceptors in the upper gastrointestinal tract that signal satiety via vagal afferents, reducing subsequent feeding bouts (Beaumont et al. 2022). From a biochemical perspective, high fiber also binds bile acids and digestive enzymes, which can reduce nutrient solubilization and slow the rate of carbohydrate and protein digestion (Rasool et al. 2023). Such changes can shift the balance of nutrient signals reaching the small intestine and ceca, with downstream effects on hormones like cholecystokinin and peptide YY that suppress appetite (van der Eijk et al. 2025).

These mechanistic interpretations extend beyond earlier descriptive reports (Sherif et al. 2025) by connecting macronutrient composition with gut physiology and neuroendocrine regulation. They support the emerging consensus in recent high‐impact studies that fiber‐mediated modulation of intake is not solely a function of physical bulk but also involves integrated neural and hormonal pathways influencing feed behavior in broilers (Rasool et al. 2023).

The observed reduction in feed intake with increasing DBRCM inclusion, particularly at the 60% level, must be interpreted through the lens of digestive physiology and nutrient signaling, not just palatability. Although sensory factors such as odor and appearance likely contributed to initial aversion, the nutrient composition and physical properties of DBRCM diets interact with gut sensory and metabolic pathways that regulate appetite. High crude fiber increases digesta viscosity and bulk, which in turn stimulates mechanoreceptors and gut hormone release (e.g., cholecystokinin and peptide YY) that signal satiety to the central nervous system (Rasool et al. 2023). Additionally, high fiber can sequester bile salts and digestive enzymes, slowing carbohydrate and protein hydrolysis in the small intestine and reducing the post‐ingestive nutrient signals that typically stimulate feeding (Kumar and Srivastava 2025). These integrated sensory and metabolic feedback mechanisms help explain why intake declines with higher DBRCM even when energy and amino acid levels are balanced.

Importantly, despite lower DMI, final body weight did not differ significantly among treatments, indicating that DBRCM diets sustained growth potential. This outcome reflects a biologically relevant compensation in nutrient utilization efficiency rather than a simple equivalence of end weights. The high biological value of blood‐derived proteins, especially rich in lysine and leucine, supports efficient muscle protein synthesis even when overall intake is lower (Potu et al. 2025). In addition, older broilers adapt digestive enzyme secretion and absorptive capacity, allowing more complete extraction of available nutrients from diets with complex protein and fiber structures. Recent studies have reported similar compensatory growth effects where diets with high‐value amino acid profiles or enzyme supplementation enable broilers to maintain growth despite reduced intake (San‐Miguel et al. 2023). This demonstrates progress beyond earlier reports that merely noted intake suppression without probing how birds biologically adjust to alternative feed substrates.

The significant depression in body weight gain (BWG) during the starter phase at high DBRCM levels underscores a well‐recognized developmental constraint in young broilers. Newly hatched chicks exhibit low digestive enzyme activity, immature mucosal surfaces, and limited hindgut fermentation capacity (Potu et al. 2025). Under such conditions, high‐fiber diets can reduce the availability of digestible amino acids and energy, particularly when protease and carbohydrase activities are insufficient to break down complex protein–fiber matrices. Reduced digestible amino acid flux from the gut lumen to the bloodstream directly limits protein deposition and growth during early life (Obafemi et al. 2024). By explicitly linking starter phase growth suppression to biochemical limitations in digestion and absorption, this work deepens understanding beyond earlier descriptive accounts and underscores the need for phase‐specific formulation strategies when integrating unconventional protein sources.

In contrast, as birds mature into the finisher phase, the convergence of BWG across treatments reflects a genuine physiological adaptation. With age, broilers exhibit increased gut surface area, enhanced secretion of digestive enzymes (including proteases and carbohydrases), and a more complex hindgut microbial community capable of fermenting fiber fractions (Wang et al. 2025). The presence of partially digested plant substrates and rumen microflora residues in DBRCM likely provided fermentable substrates for the hindgut microbiota, generating short‐chain fatty acids that serve as additional energy sources and positively influence gut health and nutrient absorption (Yitbarek and Dagnaw 2022). This adaptive response illustrates a dynamic shift in nutrient utilization efficiency over time, rather than a static outcome, and represents a significant advance relative to earlier studies that did not consider temporal changes in digestive capacity.

These findings carry several important implications for future research. First, evaluating the developmental trajectory of digestive enzymes and gut hormone profiles in broilers fed DBRCM could clarify the biochemical mechanisms of adaptation and identify optimal inclusion thresholds for each growth phase. Second, research into exogenous enzyme supplementation (e.g., cellulases or proteases) may help mitigate early‐phase digestibility constraints, reducing intake suppression without compromising economic benefits. Third, characterizing changes in hindgut microbial communities in response to DBRCM offers the potential to harness microbial fermentation pathways for improved nutrient extraction.

In sum, the present results not only confirm that moderate DBRCM inclusion supports acceptable growth performance but also illuminate the interconnected physiological, biochemical, and microbial processes that underline these outcomes. By moving beyond simple performance comparisons to mechanistic interpretation, this study aligns with recent high‐impact literature calling for a deeper understanding of how alternative feed ingredients interact with animal biology, thereby strengthening the scientific basis for sustainable poultry nutrition innovations.

The observed pattern in feed conversion ratio (FCR) further underscores the dynamic nature of how broilers physiologically adapt to DBRCM‐based diets over time. At the starter and finisher phases, higher DBRCM inclusion impaired FCR, suggesting initial inefficiencies in nutrient digestion and utilization. This may be rooted in age‐dependent differences in digestive enzyme activity: younger birds have lower endogenous protease and carbohydrase expression, which limits their ability to efficiently hydrolyze complex proteins and fiber components present in DBRCM (Beaumont et al. 2022). Likewise, the physical interactions between soluble fiber and digestive enzymes can create a viscous digesta environment that reduces enzyme–substrate contact, impairing nutrient absorption and transiently increasing maintenance energy costs relative to growth (Kumar and Srivastava 2025).

However, the absence of significant differences in overall FCR across the 56‐day cycle suggests that broilers compensate metabolically and physiologically as they mature. This compensation likely involves (i) enhanced amino acid absorption efficiency, (ii) improved nitrogen retention, and (iii) shifts in energy partitioning favoring anabolic processes over maintenance metabolism. As birds age, both brush border enzyme capacity and intestinal surface area increase, which enhances the digestion and absorption of both peptides and amino acids from complex protein matrices like DBRCM (Cohen et al. 2025). Furthermore, older birds demonstrate a more robust hindgut microbiota, increasing fermentation of fibrous components into short‐chain fatty acids that contribute additional metabolizable energy (Sherif et al. 2025). Collectively, these physiological adaptations explain how early inefficiencies associated with DBRCM can be offset over time—a process also observed in other studies on unconventional feedstuffs when diets are nutritionally balanced and age‐appropriate (Sedghi et al. 2022). From an economic standpoint, the reduction in feed cost with increasing DBRCM inclusion reflects not only cheaper ingredients but improved cost‐to‐nutrient efficiency over the full production cycle. Although the diet generated slightly higher gross revenue due to marginally greater intake, its higher feeding cost eroded net profitability. A pattern consistent with economic analyses emphasizing that net return and economic efficiency are more informative indicators than gross outputs alone (van der Eijk et al. 2025). Diets T3 and T4 optimized the balance between input costs and nutrient output, demonstrating that alternative proteins can improve economic sustainability when bird performance is maintained.

Beyond immediate economic benefits, DBRCM inclusion also contributes to sustainability goals. Valorizing slaughterhouse byproducts into feed reduces waste streams and mitigates environmental pollution associated with conventional disposal methods (e.g., anaerobic decomposition). This aligns with circular bioeconomy principles and recent sustainability research that links by‐product reutilization with reduced life‐cycle environmental impact and improved resource use efficiency in poultry systems (Rasool et al. 2023). Especially in resource‐limited production systems, strategies that simultaneously address feed scarcity, waste management, and production efficiency are of growing importance.

Nevertheless, the slight decline in palatability and potential digestibility constraints at the highest DBRCM inclusion level highlight an upper threshold beyond which physiological and sensory limitations outweigh benefits. Excessive substitution likely exacerbates the viscoelastic properties of the digesta, amplifies gut fill signals that limit intake, and dilutes the concentration of rapidly digestible nutrients, especially for young birds with immature digestive capacity (Liu et al. 2024). This observation is consistent with recent work suggesting that intermediate inclusion levels often maximize the benefits of by‐product feeds without incurring performance liabilities (Kumar and Srivastava 2025).

Importantly, the absence of significant differences in mortality, performance index, and key health indicators across treatments confirms that DBRCM diets were safe and nutritionally adequate when properly processed and balanced. This supports accumulating evidence that well‐handled animal by‐products, when integrated with appropriate amino acid and energy levels, do not compromise bird health or welfare (Rasool et al. 2023). Such confirmation strengthens confidence in the practical application of DBRCM, particularly for systems seeking to reduce reliance on high‐cost conventional protein sources without sacrificing health outcomes.

### 4.1 Implications and Future Research Directions

The present findings extend current understanding by demonstrating that DBRCM is not only an economically attractive feed ingredient but also a biologically adaptable protein source, whose utilization improves as broilers mature. This age‐dependent adaptation likely reflects the progressive development of the gastrointestinal tract, including increased digestive enzyme activity (proteases and carbohydrases), expanded absorptive surface area, and a more complex hindgut microbiota capable of fermenting fibrous rumen residues into short‐chain fatty acids, thereby enhancing energy extraction and nutrient retention (Yitbarek and Dagnaw 2022). The ability of birds to compensate for early reductions in feed intake and achieve comparable final body weights suggests that DBRCM supports efficient post‐absorptive nutrient utilization, particularly via high‐quality blood‐derived proteins that provide essential amino acids such as lysine and leucine for muscle protein accretion (San‐Miguel et al. 2023).

Future research should focus on three interconnected areas to refine the use of DBRCM in commercial broiler production. First, enzyme supplementation strategies during the starter phase could mitigate early‐phase digestibility constraints by breaking down complex protein–fiber matrices and improving amino acid availability, feed efficiency, and growth performance (Karim et al. 2026). Second, amino acid digestibility profiling across growth phases would clarify how specific essential and limiting amino acids are absorbed and utilized, informing phase‐specific formulation for optimal growth. Third, studies on gut microbiome modulation would elucidate how rumen‐derived substrates interact with microbial populations to improve fiber fermentation, short‐chain fatty acid production, and overall nutrient assimilation (Yitbarek et al. 2025). Collectively, these investigations would enable precision feeding strategies, maximizing growth performance, economic efficiency, and sustainability, while maintaining the biological and physiological integrity of the birds.

By integrating physiological, biochemical, and microbial mechanisms, these insights move beyond simple performance comparisons and highlight why DBRCM works, under what conditions it is most effective, and how it can be optimized for modern, resource‐limited poultry production systems.

## 5 Conclusion

The current research sets the foundation for dried blood–rumen content mixture (DBRCM) as a viable and reliable nonconventional protein supplement for broiler chickens. The results clearly show that DBRCM can be used at a significant concentration, substituting a substantial amount of traditional soybean meals, without compromising the health, growth, and utilization efficiency of the birds. In addition to the technical viability, the application of DBRCM has immense economic and sustainable benefits, as it reduces the dependence on expensive and human‐grade protein sources and turns the by‐products of abattoirs into valuable poultry feed. Taken together, these results place DBRCM as an economically viable, sustainable, and nutritionally adequate nonconventional protein source, especially for enhancing the profitability and sustainability of SASSO‐C44 broiler rearing.

## Author Contributions

**Demissu Hundie Senbeta:** conceptualization, supervision, validation, project administration. **Soressa Shuma:** conceptualization, supervision, project administration, validation. **Malatji Dikeledi Petunia:** conceptualization, supervision, project administration, validation.

## Funding

The authors have nothing to report.

## Ethics Statement

Ethical approval for research involving human participants (the farmers) was obtained from the Ethics Committee of the College of Environmental Sciences, South Africa University (Approval No. 2024/CAES_AREC/5937).

## Conflicts of Interest

The authors declare no conflicts of interest.

## Data Availability

Data can be obtained from the authors upon request.
